# Phosphorylation Status of Tyrosine 78 Residue Regulates the Nuclear Export and Ubiquitination of Influenza A Virus Nucleoprotein

**DOI:** 10.3389/fmicb.2019.01816

**Published:** 2019-08-07

**Authors:** Liang Cui, Weinan Zheng, Minghui Li, Xiaoyuan Bai, Wenxian Yang, Jing Li, Wenhui Fan, George Fu Gao, Lei Sun, Wenjun Liu

**Affiliations:** ^1^CAS Key Laboratory of Pathogenic Microbiology and Immunology, Institute of Microbiology, Chinese Academy of Sciences, Beijing, China; ^2^Savaid Medical School, University of Chinese Academy of Sciences, Beijing, China; ^3^Chinese National Influenza Center (CNIC), National Institute for Viral Disease Control and Prevention, Chinese Center for Disease Control and Prevention (China CDC), Beijing, China

**Keywords:** influenza A virus, nucleoprotein, phosphorylation, nuclear export, ubiquitination, CRM1

## Abstract

Phosphorylation and dephosphorylation of nucleoprotein (NP) play significant roles in the life cycle of influenza A virus (IAV), and the biological functions of each phosphorylation site on NP are not exactly the same in controlling viral replication. Here, we identified tyrosine 78 residue (Y78) of NP as a novel phosphorylation site by mass spectrometry. Y78 is highly conserved, and the constant NP phosphorylation mimicked by Y78E delayed NP nuclear export through reducing the binding of NP to the cellular export receptor CRM1, and impaired virus growth. Furthermore, the tyrosine kinase inhibitors Dasatinib and AG490 reduced Y78 phosphorylation and accelerated NP nuclear export, suggesting that the Janus and Src kinases-catalyzed Y78 phosphorylation regulated NP nuclear export during viral replication. More importantly, we found that the NP phosphorylation could suppress NP ubiquitination *via* weakening the interaction between NP and E3 ubiquitin ligase TRIM22, which demonstrated a cross-talk between the phosphorylation and ubiquitination of NP. This study suggests that the phosphorylation status of Y78 regulates IAV replication by inhibiting the nuclear export and ubiquitination of NP. Overall, these findings shed new light on the biological roles of NP phosphorylation, especially its negative role in NP ubiquitination.

## Introduction

Influenza A virus (IAV) is an enveloped virus belonging to the Orthomyxoviridae family (Kendal and Harmon, 1988). Its genome consists of eight negative sense, single-stranded RNA segments (vRNA) encoding 18 viral proteins (Chen et al., 2001; Samji, 2009; Wise et al., 2009, 2012; Jagger et al., 2012; Selman et al., 2012; Muramoto et al., 2013; Yamayoshi and Kawaoka, 2019). Each vRNA coated with multiple nucleoprotein (NP) molecules and the viral polymerase (PB2, PB1, and PA proteins) form a viral ribonucleoprotein (vRNP) complex (Zheng and Tao, 2013). Therefore, influenza A virions contain eight vRNPs which form the minimal functional units for viral transcription and replication. During the early stage of IAV infection, the nuclear import of the parent vRNP complex is primarily mediated by the nuclear localization signals (NLSs) of NP (Wu et al., 2007). Since the progeny RNP is assembled in the nucleus, each component protein requires entering the nucleus through its own NLS. Once in the nucleus, vRNPs are transcribed into viral mRNAs for the production of viral proteins and replicated into full-length complementary genomic RNA (cRNA) for amplification of vRNA and generation of progeny vRNPs. In the late phase of infection, progeny vRNPs are transported from the nucleus to plasma membrane with the assistance of the M1 and NEP proteins by CRM1-dependent nuclear export pathway (Whittaker et al., 1996a; Boulo et al., 2007; Eisfeld et al., 2015; Stevaert and Naesens, 2016).

Phosphorylation is an important posttranslational modification of IAV proteins. It has been reported that multiple sites on NP of IAV can be phosphorylated, and these phosphorylated NPs are involved in a number of activities in IAV life cycle. The phosphorylation sites on NP are predominantly serine (S), threonine (T), and tyrosine (Y). Hutchinson et al. identified 12 phosphorylated sites on NP (S9/Y10, S165, Y296/S297, S377/T378, S402/S403, S457, T472/S473) (Hutchinson et al., 2012). Among them, S165 phosphorylation had a negative effect on the viral polymerase and the dissociation of NP oligomers (Hutchinson et al., 2012; Chenavas et al., 2013; Turrell et al., 2015), and the phosphorylation and dephosphorylation of S9, Y10, and Y296 dynamically regulated the nuclear-cytoplasmic shuttling of NP (Zheng et al., 2015). In addition, the phosphorylation of S407 and S413 droved NP toward a monomeric state (Mondal et al., 2015), and the phosphorylation of S3 regulated the functionality of the N-terminal NLS of NP (Bullido et al., 2000). Also, the phosphorylation of T188 inhibited NP nuclear export and viral polymerase activity (Li et al., 2018). All above, the functions of NP phosphorylation mainly focus on the regulation of influenza virus polymerase activity and nuclear-cytoplasmic shuttling of NP, which will then affect viral replication efficiency and pathogenicity.

Apart from phosphorylation, ubiquitination is another key posttranslational modification of NP to regulate IAV replication. K184 ubiquitination of NP was reported to increase virus RNA replication and cellular deubiquitinating enzyme USP11 decreased polymerase activity and viral replication (Liao et al., 2010). Ubiquitination upregulated influenza virus polymerase function (Kirui et al., 2016). Moreover, E3 ligase TRIM22 inhibited IAV replication through inducing NP ubiquitination (Di Pietro et al., 2013), while E3 ligase CNOT4-mediated NP ubiquitination played a positive role in IAV replication (Lin et al., 2017). It has been reported that there is a cross-talk between the phosphorylation and ubiquitination (Hunter, 2007), but whether this cross-talk regulates influenza virus replication remains to be determined.

In our study, Y78 of NP was identified as a phosphorylated site. We demonstrated that the phosphorylation and dephosphorylation of Y78 regulated the virus genome replication, transcription, and NP nuclear export. Interestingly, we also found that there is a cross-talk between the phosphorylation and ubiquitination of NP.

## Materials and Methods

### Cells and Virus

The human embryonic kidney (293 T) cells, human lung alveolar epithelial (A549) cells, and Madin-Darby canine kidney (MDCK) cells (all from ATCC) were maintained in Dulbecco’s modified Eagle’s medium (DMEM, Invitrogen) with 10% fetal bovine serum (FBS, Gibco) at 37°C and 5% CO_2_. The influenza virus A/WSN/1933 (H1N1) strain was rescued using a 12-plasmid reverse genetic system (provided by Dr. Ye, Institute of Microbiology, Chinese Academy of Sciences) and propagated in 9-day-old embryonated chicken eggs (Merial, Beijing).

### Reagents and Antibodies

Phos-tag acrylamide was purchased from Wako. Dasatinib was purchased from MCE. Imatinib and AG490 were purchased from CST. An RNase inhibitor was purchased from Promega. Protease inhibitor cocktail and phosphatase inhibitor phosSTOP were purchased from Roche. Anti-FLAG (M2) Affinity Gel was purchased from Sigma-Aldrich. Pierce™ Protein G Agarose (20397) was purchased from Thermo Scientific. Rabbit polyclonal antibody against NP and mouse monoclonal antibody against M1 were generated as previously described (Liu et al., 2009). Anti-HSP70 (3A3), anti-Lamin B1 (A-11), anti-p-Tyr (sc-508), and anti-c-MYC (9E10) antibodies were purchased from Santa Cruz Biotechnology. Mouse anti-β-actin monoclonal antibody, mouse anti-GAPDH monoclonal antibody, and rabbit anti-HA antibody were purchased from Bo Ao Rui Jing (China). Mouse anti-FLAG antibody was purchased from Sigma-Aldrich. All secondary antibodies were obtained from Bai Hui Zhong Yuan Biotechnology.

### Plasmid Construction

The full-length NP sequence from the A/WSN/33 virus was cloned into the pcDNA4.0/TO, pCMV-MYC, and pCDNA3.0-FLAG vectors. FLAG or MYC-tagged NP mutants, including Y78F and Y78E were generated using a site-directed mutagenesis kit (Newpep, China). The following primers were used: NP-Y78F-up, 5′AGGAGGAATAAATTTCTAGAAGAACATCCCAGTGCGGGGA-3′; NP-Y78F-dn, 5′-ATGTTCTTCTAGAAATTTATTCCTCCTCTCGTCAAAAGCA-3′; NP-Y78E-up, 5′-AGGAGGAATAAAGAGCTAGAAGAACATCCCAGTGCGGGGA-3′; and NP-Y78E-dn, 5′-ATGTTCTTCTAGCTCTTTATTCCTCCTCTCGTCAAAAGCA-3′. The full-length TRIM22 and CRM1 were synthesized according to NCBI Reference Sequence NM_006074.5 and NM_003400.4, respectively, and then cloned into the pcDNA3.0-Flag and/or pCMV-MYC vectors. The single ubiquitin molecule was synthesized according to NCBI Reference Sequence NM_021009.6, and cloned into the pCMV-HA vector. The expression plasmids for the PA, PB1, and PB2 genes from A/WSN/33 virus were generated by cloning into the pcDNA3-FLAG vector as described previously (Wang et al., 2011). pHH21-cNS-Luc was generated by cloning cRNA promoter of NS with luciferase into pHH21 as described previously (Li et al., 2011).

### Phosphate-Affinity SDS-PAGE and Preparation for Nano-liquid Chromatography–Tandem Mass Spectrometry Analysis

IAV-infected 293 T cells were lysed in lysis buffer [150 mM NaCl, 20 mM HEPES, 1 mM EDTA (pH 7.4), 1% Triton X-100, 10% glycerol] supplemented with complete protease inhibitor cocktail and a phosphatase inhibitor phosSTOP. The NP was purified with protein G agarose beads pre-bound to a rabbit anti-NP polyclonal antibody for 3 h at 4°C. Proteins were separated by 12% Mn^2+^-Phos-tag SDS-PAGE as described previously (Wang et al., 2013). Briefly, normal polyacrylamide gel electrophoresis was conducted according to the TaKaRa protocol, with an acrylamide-pendant phosphate-tagged (Phos-tag) ligand (50 μM) and 0.1 mM MnCl_2_ (Sigma) added to the separating gel before polymerization. The gel was silver stained, and the separated bands were subjected to nano-liquid chromatography-tandem mass spectrometry (nano-LC-MS/MS) identification (LCQ Deca XP Plus; Thermo).

### Generation of Recombinant Influenza A Virus

The wild-type (WT) A/WSN/1933 (H1N1) virus and its NP mutants were generated by using a 12-plasmid-based reverse genetic system as described previously (Neumann et al., 1999; Zheng et al., 2019). Briefly, 293 T cells grown to 90% confluence in 60-mm dishes were transfected with 1 μg each of the 12 plasmids in the virus rescue system. Six hours later, the medium was replaced with DMEM plus 1 μg/ml tosylsulfonyl phenylalanyl chloromethyl ketone (TPCK)-treated trypsin. The cells were further cultured for 72 h at 37°C in 5% CO_2_, and the supernatant containing the recombinant viruses was harvested and then centrifuged at 2,000 *g* for 10 min to remove the cell debris.

### Multi-cycle Growth Curve

MDCK cells grown to monolayers in 10-cm cell culture dishes were washed with PBS and infected with virus (MOI = 0.001) for 1 h at 37°C in 5% CO_2_. The virus inoculums were removed by washing with PBS. Cell monolayers were incubated in DMEM with 1% FBS and 1 μg/ml TPCK-treated trypsin at 37°C. After infection, 500 μl of supernatant was collected at 24, 36, 48, 60, and 72 h and stored at −80°C. At the same time, 500 μl of fresh medium was added back to each dish. Finally, virus titers were determined by plaque assays as previously described (Liu et al., 2009).

### Luciferase Assay of Influenza Virus Polymerase Activity

The plasmids for expression of the PA, PB1, PB2, NP (WT or mutant) proteins, and luciferase reporter plasmids (pHH21-cNS-Luc and pcDNA-β-gal) were co-transfected into 293 T cells as previously described (Li et al., 2011). Cells transfected with the other plasmids, except the NP expression plasmid, were used as the negative control. After transfection, the cells were incubated at 37°C for 30 h, and then the amount of luciferase activity in the transfected cells was measured and normalized to the amount of β-galactosidase activity, as measured by use of standard kits (Promega, Madison, WI).

### RNA Extraction, cDNA Synthesis, and Real-Time Quantitative PCR

Total RNA was extracted from 293 T cells by use of TRIzol (Invitrogen) according to the manufacturer’s instructions. Samples were digested with DNase I and subjected to reverse transcription-PCR (RT-PCR). RNA was reverse transcribed using the following PCR primers: mRNA primer, oligo(dT); cRNA primer, 5′-AGTAGAAACAAGG-3′; and vRNA primer, 5′-AGCGAAAGCAGG-3′. A mock reaction was performed with no reverse transcriptase added to the reaction mixture. The analysis of relative M1 gene expression was performed using the following PCR primers: M1-up (5′-TCTGATCCTCTCGTCATTGCAGCAA-3′) and M1-dn (5′-AATGACCATCGTCAACATCCACAGC-3′). GAPDH served as an internal control, using the PCR primers GAPDH-up (5′-GGTGGTCTCCTCTGACTTCAACA-3′) and GAPDH-dn (5′-GTTGCTGTAGCCAAATTCGTTGT-3′). The cycling conditions comprised an initial denaturation step of 30 s at 95°C, followed by 40 two-step cycles (95°C for 5 s and 60°C for 31 s). Dissociation curve analysis was performed after each assay to ensure specific target detection.

### Immunoprecipitation and Western Blotting Analyses

293 T cells were lysed in lysis buffer [150 mM NaCl, 20 mM HEPES, 1 mM EDTA (pH 7.4), 1% Triton X-100 and 10% glycerol], supplemented with complete protease inhibitor cocktail and phosphatase inhibitor phosSTOP. After an incubation period of 30 min at 4°C, insoluble components were removed by centrifugation at 12,000 *g* for 15 min. Lysates were incubated with anti-FLAG M2 affinity gel for 2 h. Following four washes in wash buffer [300 mM NaCl, 20 mM HEPES, 1 mM EDTA (pH 7.4), 1% Triton X-100 and 10% glycerol], the precipitated proteins were separated by SDS-PAGE and then transferred to immobilon polyvinylidene difluoride (PVDF) membranes (Millipore Corporation, Billerica, MA). The membranes were blocked for 2 h at room temperature in blocking solution (5% skim milk powder, 1% BSA and 0.5% Tween 20 in PBS), and proteins were detected using appropriate antibodies, followed by the addition of anti-rabbit or anti-mouse secondary antibody coupled to horseradish peroxidase. Proteins were visualized by use of chemiluminescence detection reagents.

### Indirect Immunofluorescence Assay

Indirect immunofluorescence assays (IFAs) were performed with a Leica SP8 confocal laser scanning microscope. Coverslips carrying the cells were washed with PBST (PBS plus 1% Triton X-100) and fixed with 4% paraformaldehyde. Cells were then blocked with 4% bovine serum albumin (BSA) dissolved in PBST and stained with anti-NP and anti-FLAG antibodies. Secondary antibodies were tetramethyl rhodamine isocyanate (TRITC)-conjugated anti-rabbit IgG and fluorescein isothiocyanate (FITC)-conjugated anti-mouse IgG. Finally, the location of cell nucleus was labeled with 4′,6-diamidino-2-phenylindole (DAPI). The wavelengths of TRITC, FITC, and DAPI are 561 nm, 488 nm, and 405 nm.

### The Separation of Nuclear and Cytoplasmic Fractions

293 T cells in 6-cm dishes were harvested into 300 μl of CLB buffer (10 mM NaCl, 1 mM KH_2_PO_4_, 10 mM HEPES, 5 mM NaHCO_3_, 1 mM CaCl_2_, 0.5 mM MgCl_2_, 5 mM EDTA, and Complete protease inhibitor cocktail). The cells were allowed to swell for 5 min on ice and then dounce-homogenized 50 times. After centrifugation at 7,500 rpm for 2 min at 4°C, the pellets represented nuclei plus debris, and the supernatants were cytosol plus plasma membrane. The nucleus-debris pellet was then resuspended in 1 ml of TSE buffer [10 mM Tris (pH 7.5), 300 mM sucrose, and 1 mM EDTA] and dounce-homogenized 30 times, followed by centrifugation at 5,000 rpm for 2 min. The pellet was resuspended and washed twice to obtain the final nuclear pellet. The final pellet was resuspended in 30 μl of TSE buffer.

### Protein Degradation Assay

293 T cells were transfected with pcDNA3.0-FLAG-NP-WT plasmid or pcDNA3.0-FLAG-NP mutant (Y78F, Y78E) plasmids or MYC-TRIM22 plasmid. The cells were harvested at 30 h after transfection. Western blotting was probed with anti-NP, anti-c-MYC, and anti-β-actin antibodies.

### Ubiquitination Assay

293 T cells were transfected with expression constructs for FLAG-tagged NPs and HA-tagged ubiquitin or MYC-TRIM22. Thirty-six hours post-transfection, cell extracts were then immunoprecipitated with FLAG-M2 beads. The eluted proteins were analyzed by western blotting using an anti-HA, anti-FLAG, and c-MYC antibodies.

### Statistical Analyses

Sequence alignments were performed using MegAlign software (DNAstar Software, San Diego, CA). Statistical analyses were performed using Prism6 software (GraphPad Software, San Diego, CA).

## Results

### Nucleoprotein Y78 Is a Highly Conserved Phosphorylation Site of Influenza A Virus

**Figure 1:**
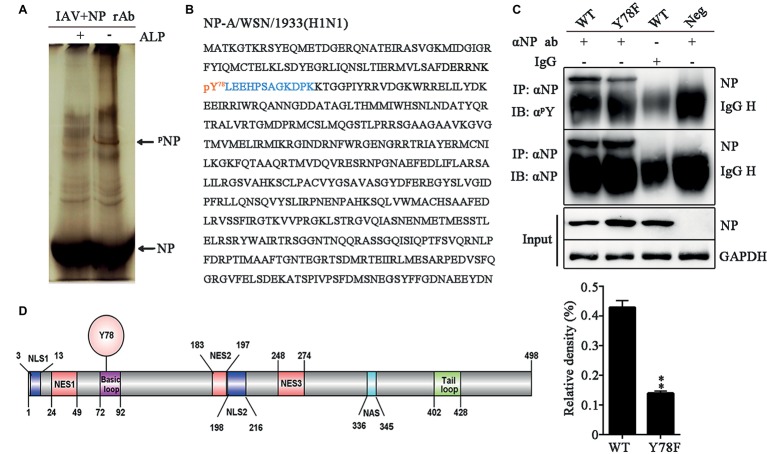
NP Y78 is a conserved phosphorylation site. **(A)** The Phos-tag SDS-PAGE gel of phosphorylated NP immunoprecipitated with rabbit anti-NP antibody. The IAV (WSN)-infected 293 T cells were lysed in lysis buffer supplemented with complete protease inhibitor cocktail and a phosphatase inhibitor phosSTOP. The extra band in untreated cells was considered to be a phosphorylated band compared to alkaline phosphatase (ALP)-treated cells. The band was digested and subjected to LC-MS/MS analysis. The locations of NP and phosphorylated NP bands (^p^NP) are indicated by arrows. **(B)** LC-MS/MS analysis of the phosphorylated band. The band was identified as the IAV NP. The identified polypeptide sequence is indicated in blue, and the phosphorylated tyrosine site is indicated in red. **(C)** Detection of tyrosine-phosphorylated NP. A549 cells infected with viruses (WT or Y78F WSN, MOI = 1) were lysed at 12 h.p.i. and then were incubated with NP antibody and protein G agarose beads. The uninfected cells immunoprecipitated with anti-NP antibody and the infected cells immunoprecipitated with non-specific IgG served as controls. The immunoprecipitated NPs of the WT and Y78F WSN viruses were detected using anti-NP antibody (αNP) or anti-p-Tyr antibody (α^p^Y) (top). The relative density of phosphorylated NP was normalized to total NP (below). Data are shown as mean + SD (*n* = 3). Difference between WT and Y78F mutant viruses was tested using unpaired Student’s *t*-test. ^**^p < 0.01. **(D)** Schematic diagram of NP functional domains, including Y78, nuclear localization sequence (NLS), nuclear export sequence (NES), nuclear aggregation sequence (NAS), basic loop and tail loop region.

To identify the phosphorylation site of IAV NP, 293 T cells were infected with the influenza virus A/WSN/1933 (H1N1) (WSN) and NP was purified by protein G agarose beads pre-bound to rabbit anti-NP polyclonal antibody. Phos-tag SDS-PAGE was used to purify the phosphorylated NP (Kinoshita et al., 2009). After silver staining, an additional band was found in the purified NP lane compared to the alkaline phosphatase (ALP)-treated lanes (Figure 1A). Then, this band was cut from the gel and sent for mass spectrometric identification. LC-MS/MS analysis showed that this band was in fact NP of WSN and the tyrosine 78 residue (Y78) was a potential phosphorylation site (Figure 1B). To further confirm whether Y78 can be phosphorylated during virus infection, Y was mutated to phenylalanine (F) to mimic the dephosphorylated state of this site. The wild type (WT) and NP Y78F mutant viruses were rescued by the 12-plasmid reverse genetic system and used to infect A549 cells. At 12 h post-infection (h.p.i.), the cell lysate was enriched with protein G agarose beads pre-bound to anti-NP antibody. The levels of phosphorylated NP were evaluated by use of anti-phosphor-tyrosine antibody (α^p^Y). We found that the phosphorylation level of NP Y78F mutant was greatly decreased compared with that of WT NP (Figure 1C), demonstrating that NP Y78 could be phosphorylated during virus infection. Collectively, only Y78 was identified as a phosphorylation site of NP in this study.

The schematic diagram of NP functional domains demonstrated that Y78 belongs to the basic loop region (residues 72-92) (Figure 1D). At the same time, the full-length NP sequences of IAV isolates from the Influenza Virus Sequence Database of NCBI were analyzed by multiple sequence alignment (Table 1). The results indicate that Y78 is highly conserved.

**Table 1:** Conservation of Y78 among NPs of different subtypes of influenza A virus.

Numbers of isolates	H1N1 500	H2N2 108	H3N2 500	H5N1 628	H7N1 69	H7N2 84	H7N3 192	H7N7 119	H7N9 198	H9N2 862	H10N8 38
Conservation (%)	99.6	100.0	99.8	100.0	100.0	100.0	100.0	100.0	100.0	99.9	100.0

*The full-length NP sequences among all the isolates of influenza A virus from the Influenza Virus Sequence Database of NCBI were analyzed by multiple sequence alignment*.

### Effects of Nucleoprotein Y78 Phosphorylation on Viral Replication

**Figure 2:**
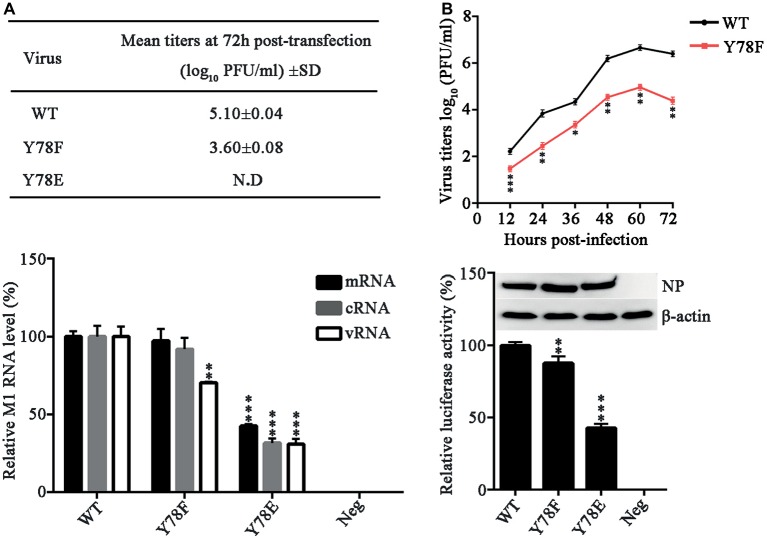
NP Y78 phosphorylation affects IAV replication and polymerase activity. **(A)** Rescue of WT and Y78 mutant WSN viruses using the 12-plasmid reverse genetic system. The supernatants of 293 T cells were harvested at 72 h.p.t. and used for plaque assays in MDCK cells. N.D represents failed rescue; SD represents standard deviation. **(B)** Multi-cycle growth curves of viruses. A549 cells were respectively infected with WT and Y78F mutant WSN viruses (MOI = 0.001). Then the supernatants of virus-infected cells were examined by plaques assays at different time points. Data are shown as mean + SD (*n* = 3). Difference between WT and Y78F mutant viruses was tested using unpaired Student’s *t*-test. ^*^p < 0.05; ^**^p < 0.01; ^***^p < 0.001. **(C)** The expression levels of M1 RNAs (mRNA, cRNA, and vRNA) were tested by real-time PCR. 293 T cells were transfected with the 12-plasmid reverse genetic system containing NP WT, Y78E, Y78F, or empty vector (Neg) plasmid for 48 h. Data are shown as mean + SD (*n* = 3). Differences between mutant and WT NPs were evaluated using one-way analysis of variance (ANOVA) followed by Dunnett’s test. ^**^p < 0.01; ^***^p < 0.001. **(D)** The polymerase activity of WT and mutant NPs were determined by luciferase assays. Luciferase activity was measured at 30 h after transfection of viral proteins (PB1, PB2, and PA); β-gal; and the cNS-Luc plasmid in 293 T cells. Differences of luciferase activities between the mutant and WT NPs were evaluated using one-way ANOVA followed by Dunnett’s test (below). Data are shown as mean + SD (*n* = 3). ^**^p < 0.01; ^***^p < 0.001, and the expressions of NPs were analyzed by western blotting (top).

To investigate the role of NP Y78 phosphorylation in influenza virus replication and packaging, we attempted to rescue the WT and mutant recombinant WSN viruses. We introduced glutamate (E) substitution at Y78 to mimic the constitutively phosphorylated residue and F at Y78 to mimic the constitutively dephosphorylated residue. The 12-plasmid reverse genetic system containing WT or mutant NP plasmid was transfected into 293 T cells to rescue the WT or NP mutant virus, and the supernatants were harvested at 72 h post-transfection (h.p.t.) for plaque assays. In three independent viral packaging experiments, both the WT and Y78F WSN viruses were successfully rescued, and the titer of Y78F mutant virus was greatly reduced compared with that of WT virus (Figure 2A). However, the Y78E mutation did not yield any infectious virus. These results suggested that the Y78E mutation was lethal to the virus and might have an important impact on virus assembly. To further evaluate the effect of NP Y78 dephosphorylation on the growth kinetics of IAV, we examined the multi-step growth curves of WT and Y78F viruses in A549 cells. We found that the virus titers of Y78F mutant were reduced to 10~100-fold compared with those of WT virus (Figure 2B), indicating that non-phosphorylated NP Y78F decreased the replication efficiency of IAV. Together, our results suggest that the phosphorylation and dephosphorylation of NP Y78 are both vital for IAV replication.

NP is required for viral RNA synthesis (Marklund et al., 2012), and binds to viral RNA segments to form vRNPs (Portela and Digard, 2002). To examine whether the expression of vRNA, cRNA, and mRNA is affected by NP Y78 phosphorylation, 293 T cells were transfected with the 12-plasmid reverse genetic system expressing WT, Y78F, or Y78E mutant NP. The relative quantities of M1 RNAs (mRNA, cRNA, and vRNA) were determined by real-time PCR. We found that the Y78E mutation remarkably reduced the levels of all three types of M1 RNAs, while the Y78F mutation only decreased the level of M1 vRNA (Figure 2C).

In addition, NP has long been recognized as the second most abundant protein of influenza virus and an important component of the vRNP complexes. It plays a major role in the overall structural organization and stabilization of the complexes, then affects the polymerase activity of vRNP (Ruigrok and Baudin, 1995). Therefore, a luciferase assay was performed to explore the effects of Y78 phosphorylation on vRNP functionality. As shown in Figure 2D, both Y78E and Y78F mutations resulted in a significant reduction of relative luciferase activity, suggesting that Y78E and Y78F mutations reduced the amount of functional vRNP. Altogether, continuous phosphorylation and dephosphorylation of Y78 mimicked by Y78E and Y78F mutant are both disadvantageous to virus replication.

### Phosphorylation Status of Y78 Regulates the Nuclear Export of Nucleoprotein

**Figure 3:**
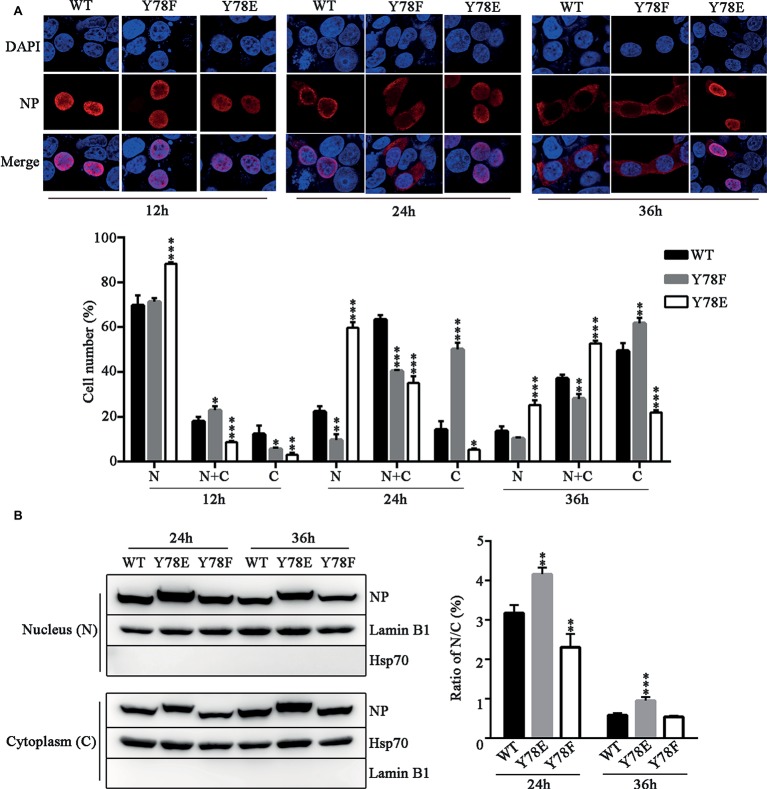
Y78 phosphorylation inhibits the nuclear export of NP. **(A)** The cellular localizations of WT and mutant NPs (Y78F and Y78E) were determined using IFAs. 293 T cells were transfected with plasmids expressing WT or mutant NPs. Cells were fixed at 12, 24, and 36 h.p.t. The nucleus was stained with DAPI (blue), and the subcellular distribution of NP (red) was analyzed (top). At least 600 cells from each condition were scored as predominantly nuclear (N), nuclear and cytoplasmic (N + C), or predominantly cytoplasmic (C) (below), and the percentage of cells representing each is shown. Differences of cellular localization between the mutant and WT NPs were evaluated using one-way ANOVA followed by Dunnett’s test. Data are shown as mean + SD (*n* = 3). ^*^p < 0.05; ^**^p < 0.01; ^***^p < 0.001. **(B)** Immunoblot analysis of the subcellular location of NP. 293 T cells were transfected with plasmid expressing WT, Y78F, or Y78E NP. The nuclear and cytoplasmic fractions were separated at 24 and 36 h.p.t. and detected using anti-NP and anti-Lamin B1 or anti-Hsp70 antibody (left). Nuclear NP was normalized to Lamin B1, and cytoplasmic NP was normalized to Hsp70. Data are shown as mean + SD (*n* = 3). The differences of N/C ratio between mutant and WT NPs were evaluated using one-way ANOVA followed by Dunnett’s test (right). ^**^p < 0.01; ^***^p < 0.001.

The dynamic cellular localization of NP is critical for efficient viral replication and transcription (Ozawa et al., 2007), and NP phosphorylation regulates its nuclear-cytoplasmic shuttling (Zheng et al., 2015). Therefore, we examined the intracellular localization of NP mutants by indirect immunofluorescence assays (IFAs). The WT and mutant NP (Y78F, Y78E) plasmids were transfected into 293 T cells respectively; then, the cellular localization of NP was observed at different time points. We found that the WT NP mainly existed in the nucleus at 12 h.p.t., transferred to the periphery of the inner region of the nucleus and cytoplasm at 24 h.p.t., then completely exported to the cytoplasm at 36 h.p.t. In contrast, Y78F mutant exhibited nuclear localization at 12 h.p.t., and distributed in the cytoplasm at 24 and 36 h.p.t, while Y78E mutant was located in nucleus at all time points (Figure 3A). Moreover, the cell counting data also clearly demonstrated that Y78E mutation resulted in the nuclear retention of NP and Y78F mutation promoted the nuclear export of NP. Furthermore, the nuclear and cytoplasmic locations of WT and mutant NPs were analyzed by western blotting. The results showed that the nuclear/cytoplasm (N/C) ratio of Y78E mutant NP was increased compared with that of WT NP at 24 and 36 h.p.t., while the N/C ratio of Y78F mutant NP was lower than that of WT NP at 24 h.p.t. and similar to that of WT NP at 36 h.p.t. (Figure 3B), which are consistent with the IFA results. Collectively, the phosphorylation and dephosphorylation of Y78 mimicked by Y78E and Y78F mutants regulate the nuclear export of NP.

**Figure 4:**
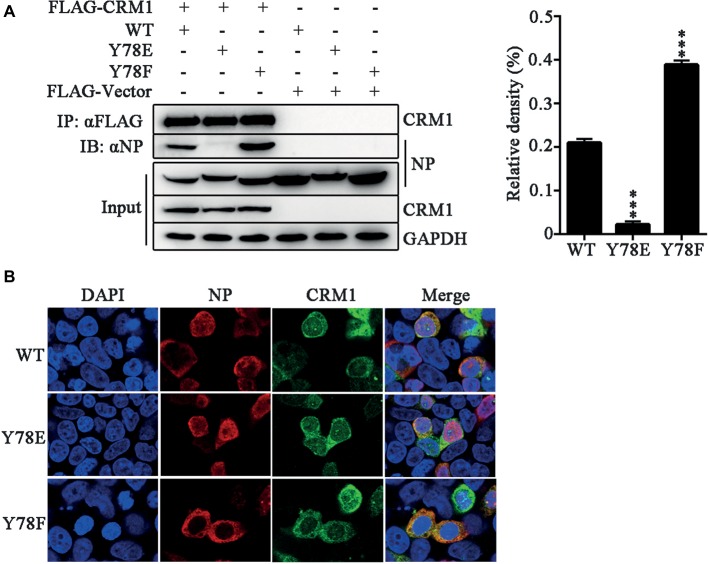
Y78 phosphorylation negatively regulates the binding of NP to CRM1. **(A)** Co-immunoprecipitation (CO-IP) experiments of WT and mutant NPs with CRM1. The NP (WT, Y78F, and Y78E) with pcDNA4.0/TO-tagged plasmid and pCDNA3.0-FLAG-CRM1 plasmid were co-transfected into 293 T cells, and cells were lysed at 24 h.p.t. FLAG-CRM1 was immunoprecipitated with anti-FLAG agarose, and the associated NP was detected using rabbit anti-NP antibody (left). The relative density of immunoprecipitated NPs was normalized to immunoprecipitated CRM1 (right). Data are shown as mean + SD (*n* = 3). Differences between mutant and WT NPs were evaluated using one-way ANOVA followed by Dunnett’s test. ^***^p < 0.001. **(B)** The co-localizations of CRM1 with NPs were examined by IFAs. The 293 T cells expressing FLAG-CRM1 and pCDNA4.0-NP (WT, Y78F, and Y78E) were fixed and stained with anti-NP (red) and anti-FLAG (green) antibodies at 24 h.p.t. The nucleus was stained with DAPI (blue).

Previous studies have suggested that a classical cytoplasmic receptor protein CRM1 can interact with NP and transport NP into the cytoplasm (Elton et al., 2001; Yu et al., 2012). To detect whether Y78 phosphorylation affects NP nuclear export by inhibiting the interaction between NP and CRM1, the FLAG-CRM1 and pCDNA4.0-NP (WT, Y78F and Y78E) plasmids were co-transfected in 293 T cells and their interactions were determined by co-immunoprecipitation (CO-IP) assays. As expected, CRM1 could interact with the WT and Y78F NPs, but hardly interacted with Y78E NP. Moreover, the binding of Y78F to CRM1 was stronger than that of WT NP to CRM1 (Figure 4A). At the same time, to examine the effect of Y78 phosphorylation on the co-localization of NP and CRM1, we performed IFAs in 293 T cells expressing CRM1 and NPs. We found that CRM1 displayed a stronger co-localization with Y78F NP than with WT NP in the cytoplasm and a very weak co-localization with Y78E mutant NP (Figure 4B). All data indicate that the phosphorylation and dephosphorylation of Y78 can alter the binding affinity of NP-CRM1.

**Figure 5:**
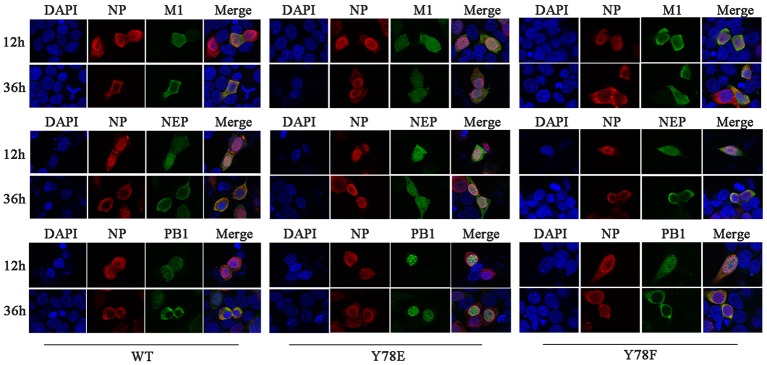
Y78 phosphorylation status regulates the nuclear export of NEP-M1-vRNP complex. The co-localizations of NP with M1/NEP/PB1 were examined by IFAs. The 12-plasmid reverse genetic system containing WT, Y78F, or Y78E NP plasmid was transfected into 293 T cells. At 12 and 36 h.p.t., cells were fixed and stained with anti-NP (red) and anti-M1/NEP/PB1 antibodies (green). The nucleus was stained with DAPI (blue).

The nuclear export of vRNP complex is mediated by the CRM1-NEP-M1-vRNP complex, during which the nuclear transport capacity of the NEP and M1 plays an important role (Fukuda et al., 1997; O’Neill et al., 1998; Huang et al., 2013). Moreover, NP bridges the CRM1-NEP-M1 complex and viral polymerase heterotrimer during vRNP nuclear export (Akarsu et al., 2003; Shimizu et al., 2011). Therefore, we next investigate whether NP Y78 phosphorylation status could further affect the nuclear export of NEP-M1-vRNP complex. IFAs were used to examine the cellular co-localization of NP and M1/NEP/PB1. Because the Y78E NP mutant recombinant virus could not be generated, the 12-plasmid reverse genetic packaging system containing WT, Y78F, or Y78E NP plasmids was transfected in 293 T cells to mimic the replication process of eight-segmented genome. The results showed that Y78F NP and M1/NEP/PB1 were co-localized in the nucleus at 12 h.p.t. and in the cytoplasm at 36 h.p.t., which was similar with the co-localization of WT NP and M1/NEP/PB1. However, the Y78E mutant NP and M1/NEP/PB1 were co-localized in the nucleus at all time points (Figure 5), indicating that the Y78E mutant might further block the nuclear export of NEP-M1-vRNP complex. Altogether, these results suggest that the phosphorylation status of Y78 might regulate the nuclear export of NEP-M1-vRNP complex in addition to non-RNP NPs by altering the binding affinity of NP-CRM1.

### Janus and Src Kinases Catalyze Y78 Phosphorylation and Regulate Nucleoprotein Nuclear Export During Viral Replication

**Figure 6:**
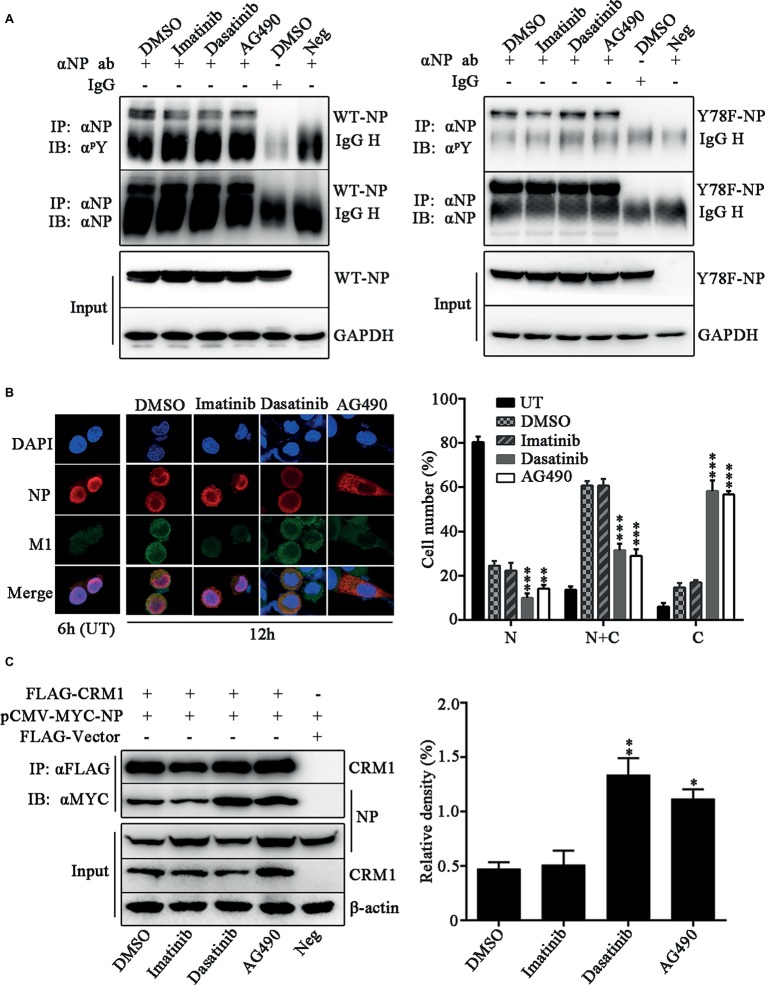
Janus and Src kinases are involved in Y78 phosphorylation-regulated NP nuclear export. **(A)** Effect of tyrosine kinase inhibitors on NP phosphorylation. A549 cells were infected with WSN or Y78F mutant virus (MOI = 1) for 12 h and then treated with the tyrosine kinase inhibitors Imatinib (10 μM), Dasatinib (10 μM), and AG490 (50 μM) for 6 h, with DMSO as a control (the inhibitors were dissolved in DMSO). Then cells were lysed and incubated with NP antibody and protein G agarose beads. The uninfected cells immunoprecipitated anti-NP antibody and the infected cells that were treated with DMSO and immunoprecipitated with non-specific IgG served as controls. The immunoprecipitated NPs were detected using anti-NP antibody (αNP) or anti-p-Tyr antibody (α^p^Y). **(B)** Effect of tyrosine kinase inhibitors on the nuclear export of NP. A549 cells were infected with WSN (MOI = 0.1) and treated with CHX (100 μg/ml) and several tyrosine kinase inhibitors, including Imatinib (10 μM), Dasatinib (10 μM) and AG490 (50 μM) for 2 h at 10 h.p.i., with DMSO as a control. Cells were fixed and stained with anti-NP (red) antibody. The nucleus was stained with DAPI (blue). At the same time, some cells only infected with WSN were fixed at 6 h.p.i. (left). At least 600 cells from each group were scored as N, N + C, or C, and the percentage of cells representing each is shown. Data are shown as mean + SD (*n* = 3). Differences of cellular localization between the tyrosine kinase inhibitor- and DMSO-treated groups were evaluated using one-way ANOVA followed by Dunnett’s test (right). ^**^p < 0.01; ^***^p < 0.001. **(C)** Effects of tyrosine kinase inhibitors on the binding of NP to CRM1. 293 T cells were co-transfected FLAG-CRM1 and MYC-NP plasmids, then tyrosine kinase inhibitors Imatinib (10 μM), Dasatinib (10 μM), or AG490 (50 μM) were added to medium at 24 h.p.t., with DMSO as a control. Cells were lysed for CO-IP assays after 6 h post inhibitor treatment (left). The relative density of immunoprecipitated NPs was normalized to immunoprecipitated CRM1 (right). Data are shown as mean + SD (*n* = 3). Differences of NP-CRIM1 interaction between the tyrosine kinase inhibitor- and DMSO-treated groups were evaluated using one-way ANOVA followed by Dunnett’s test. ^*^p < 0.05; ^**^p < 0.01.

We further investigated which kinds of tyrosine kinases play the dominant role in Y78 phosphorylation and their effect on NP sub-cellular localization. The tyrosine kinase inhibitors, including Imatinib (Bcr-Abl inhibitor), Dasatinib (Src inhibitor), and AG490 (JAK inhibitor), were used to treat WSN or Y78F mutant virus-infected cells. We detected the effect of these three tyrosine kinase inhibitors on the tyrosine phosphorylation level of WT and Y78F NP in A549 cells. We found that all of these three tyrosine kinase inhibitors decreased the tyrosine phosphorylation of WT NP (Figure 6A, left). Moreover, Imatinib significantly inhibited phosphorylation of Y78F NP, indicating that Imatinib had influence on the phosphorylation of other tyrosine sites rather than Y78. However, Dasatinib and AG490 could hardly affect the phosphorylation of Y78F NP, suggesting that Janus and Src kinases were involved in Y78 phosphorylation (Figure 6A, right). Furthermore, IFAs were performed to examine the effect of these inhibitors on NP localization during viral infection. A549 cells were infected with WSN and treated with CHX (100 μg/ml) and tyrosine kinase inhibitors. Our data showed that NP was located in the nucleus at 6 h.p.i. However, at 12 h.p.i., only a small portion of NP distributed in the cytoplasm of DMSO- and Imatinib-treated cells. Nevertheless, NP was mainly localized in the cytoplasm in Dasatinib and AG490-treated cells (Figure 6B). Thus, the Src and JAK family kinases delayed NP nuclear export, whereas the Bcr/Abl family kinases might be irrelevant to NP nuclear export. Besides, the cell counting data also provided evidence that Dasatinib and AG490 accelerated the nuclear export of NP. To further elucidate the mechanism by which these tyrosine kinase inhibitors affect NP nuclear export, CO-IP assays were performed to examine the effect of these tyrosine kinase inhibitors on the binding of NP to CRM1. 293 T cells were co-transfected with FLAG-CRM1 and MYC-NP plasmids, then treated with tyrosine kinase inhibitors or DMSO. It was indicated that the interaction between NP and CRM1 was significantly enhanced with the treatment of Dasatinib or AG490 rather than DMSO or Imatinib (Figure 6C). Collectively, the tyrosine kinase Src and JAK family kinases-catalyzed Y78 phosphorylation decreases the interaction between NP and CRM1, and then suppresses NP nuclear export.

### Y78 Phosphorylation Regulates Nucleoprotein Ubiquitination

**Figure 7:**
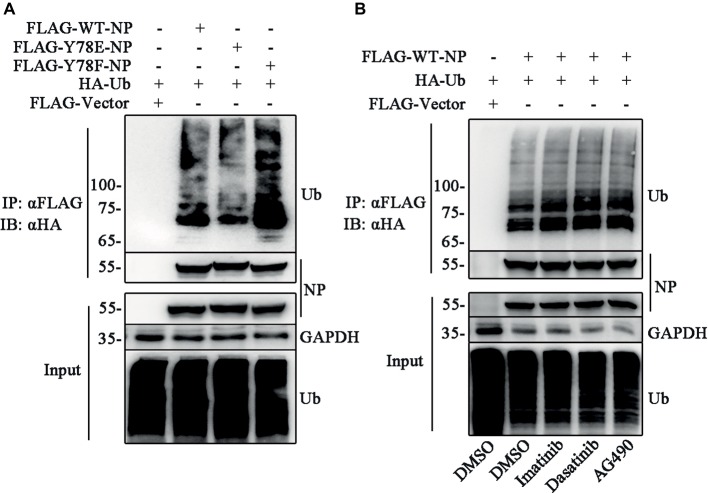
Y78 phosphorylation inhibits NP ubiquitination. **(A)** Effect of Y78 phosphorylation on NP ubiquitination. 293 T cells were transfected with FLAG-tagged NPs and HA-tagged ubiquitin (HA-Ub). Cell extracts were immunoprecipitated with anti-FLAG beads at 30 h.p.t., and the ubiquitination was detected using rabbit anti-HA antibody. **(B)** Effect of tyrosine kinase inhibitors on NP ubiquitination. 293 T cells were transfected with FLAG-tagged WT NP and HA-tagged ubiquitin (HA-Ub). At 30 h.t.p., cells were treated with DMSO or inhibitors for 6 h. Cell extracts were immunoprecipitated with anti-FLAG beads and the ubiquitination was detected using rabbit anti-HA antibody.

Several lines of evidence indicate that the phosphorylation-mediated subcellular localization of substrates regulates the ubiquitination (Besson et al., 2006; Hunter, 2007). Thus, we try to investigate the relationship between NP Y78 phosphorylation and NP ubiquitination. The ubiquitination assays were performed to detect whether Y78 phosphorylation affected NP ubiquitination. 293 T cells were co-transfected with FLAG-NP (WT, Y78F, and Y78E) and HA-ubiquitin (HA-Ub) plasmids, followed by immunoprecipitation with anti-FLAG beads. Compared to the ubiquitination of WT NP, the ubiquitination of Y78F NP was slightly increased, while the ubiquitination of Y78E NP was greatly reduced (Figure 7A), indicating that the phosphorylation status of Y78 affected NP ubiquitination. At the same time, we further explored the effect of tyrosine kinase-catalyzed NP phosphorylation on NP ubiquitination. 293 T cells were co-transfected with FLAG-WT-NP and HA-Ub plasmids, then treated with tyrosine kinase inhibitors (Imatinib, Dasatinib, and AG490) or DMSO, followed by immunoprecipitation with anti-FLAG beads. The ubiquitination of NP was significantly increased when treated with these three inhibitors, especially Dasatinib and AG490 (Figure 7B), suggesting that Y78 phosphorylation is important for regulating NP ubiquitination.

### Phosphorylation Status of Y78 Regulates TRIM22-Mediated Nucleoprotein Ubiquitination

**Figure 8:**
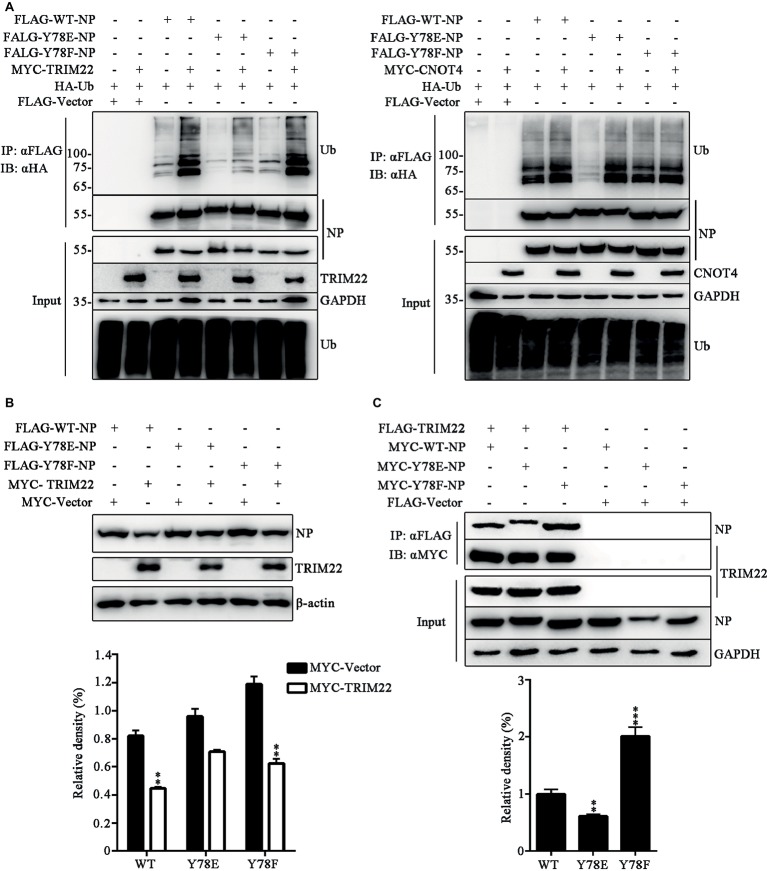
Y78 phosphorylation regulates TRIM22-mediated NP ubiquitination. **(A)** Effect of TRIM22 and CNOT4 on the ubiquitination of NPs. Immunoblot analysis of lysates in 293 T cells transfected with various combinations of plasmids for 30 h, followed by immunoprecipitation with anti-FLAG beads. **(B)** Effect of TRIM22 on the stability of IAV NPs. 293 T cells were transfected with FLAG-NP (WT, Y78F, or Y78E) plasmid, along with MYC-TRIM22 or an empty vector for 30 h. Cells were lysed and then detected with corresponding antibodies (top). The relative density of NPs was normalized to β-actin (below). Data are shown as mean + SD (*n* = 3). Differences of NP stability between the TRIM22- and Vector-transfected groups were tested using unpaired Student’s *t*-test. ^**^p < 0.01. **(C)** Effect of Y78 phosphorylation on the interaction of TRIM22 with WT or mutant NPs. 293 T cells were transfected with MYC-NP (WT, Y78F, and Y78E) and FLAG-TRIM22 and lysed at 30 h.p.t. FLAG-TRIM22 was immunoprecipitated with anti-FLAG beads, and the associated NPs were detected using rabbit anti-NP antibody (top). The relative density of immunoprecipitated NPs was normalized to immunoprecipitated TRIM22 (below). Data are shown as mean + SD (*n* = 3). Differences between mutant and WT NPs were evaluated using one-way ANOVA followed by Dunnett’s test. ^**^p < 0.01; ^***^p < 0.001.

It has been established that the E3 ligase TRIM22 mediates ubiquitin-proteasome degradation of NP (Di Pietro et al., 2013), and CNOT4 is also a ubiquitin ligase of NP, but CNOT4-mediated ubiquitination does not lead to NP degradation (Lin et al., 2017). Here, we explored the effect of phosphorylation of the WT and mutant NPs on TRIM22- and CNOT4-mediated ubiquitination in 293 T cells transfected with various plasmids. It was found that TRIM22 enhanced the ubiquitination of all these three types of NPs, and the TRIM22-mediated ubiquitination of Y78E NP was much weaker than that of WT and Y78F NPs (Figure 8A, left). In contrast, Y78E and Y78F mutations had no significant effect on CNOT4-mediated NP ubiquitination, compared with WT NP (Figure 8A, right). These results suggested that Y78 phosphorylation decreased TRIM22-mediated NP ubiquitination. Next, we sought to examine the effect of Y78 phosphorylation on TRIM22-mediated stability of NP. 293 T cells were transfected with NP (WT, Y78F, or Y78E), along with or without TRIM22. The western blotting results showed that TRIM22 could promote the degradation of WT and Y78F NPs, but not that of Y78E (Figure 8B), indicating that Y78 phosphorylation inhibited TRIM22-mediated NP degradation.

E3 ligases usually interact with substrates, so we wondered whether Y78 phosphorylation affected NP ubiquitination by regulating the binding of NP to TRIM22. The interaction between NP (WT, Y78F, and Y78E) and TRIM22 was determined by CO-IP assays. 293 T cells were transfected with MYC-NPs and FLAG-TRIM22. We found that the Y78E mutant reduced the binding of NP to TRIM22, while the Y78F mutant enhanced NP-TRIM22 interaction (Figure 8C). The above results suggest that the phosphorylation status of Y78 might regulate the ubiquitin-mediated proteasome degradation of NP by changing the interaction between NP and TRIM22.

## Discussion

As two types of the posttranslational modifications in eukaryotic biology, phosphorylation and ubiquitination can take a cross-talk (Swaney et al., 2013). Phosphorylation can positively or negatively regulate ubiquitination of the same protein and the cross-talk between phosphorylation and ubiquitination is pivotal in numerous cellular processes. For example, phosphorylation decreases ubiquitination of the thiazide-sensitive cotransporter NCC in endocytosis (Rosenbaek et al., 2014). Phosphorylation of membrane-associated cyclin Y by CDK14 triggers its ubiquitination and degradation (Li et al., 2014). The mineralocorticoid receptor phosphorylation affects its ubiquitylation and inhibits the aldosterone-mediated degradation (Faresse et al., 2012). However, the relationship between phosphorylation and ubiquitination of influenza virus protein has not been determined yet. Our study demonstrated that IAV NP Y78 phosphorylation reduced NP ubiquitination, which shed new light on the biological roles of NP phosphorylation. It has been reported that phosphorylation can regulate ubiquitination by creating phosphordegrons to promote recognition by an E3 ligase, or regulating the E3 ligase activity, deubiquitinating enzyme activity, and substrate/ligase interaction at the level of subcellular compartmentalization (Hunter, 2007). The E3 ubiquitin ligase activity of TRIM22 is shown to be responsible for the degradation of the IAV NP, which is essential for the replication of IAV (Di Pietro et al., 2013), and of the human Hepatitis C NS5A (Yang et al., 2016). In this study, we found that NP Y78 phosphorylation could negatively regulate ubiquitination by impairing the binding of NP to TRIM22. It is likely that NP Y78 phosphorylation is able to decrease the NP-TRIM22 interaction directly by changing the conformation of NP. Otherwise, since TRIM22 is a molecule localized in the cytoplasm, it is natural that NP Y78 phosphorylation indirectly decreases the contact frequency of TRIM22 and NP by blocking NP nuclear export.

Phosphorylation and dephosphorylation is a reversible dynamic equilibrium process. IAV NP underwent phosphorylation and dephosphorylation at various stages during viral multiplication, and the exposed NP phosphorylation sites might also be different during various infection periods (Kistner et al., 1989). In the present study, continuous phosphorylation and dephosphorylation of Y78 mimicked by Y78E and Y78F mutant are both disadvantageous to virus replication. We believe that this is a regulatory strategy for influenza viruses to proliferate in host cells. If the phosphorylation site of NP is overly phosphorylated or dephosphorylated, the intracellular kinases or phosphatases also need to be continuously activated, causing disorder of cell signal transmission and a series of defense mechanisms against the virus, such as cell death, autophagy, etc., which is harmful to both cell and virus. Thus, the opportune phosphorylation status (phosphorylation or dephosphorylation) of various phosphorylation sites is critical for virus replication. For instance, during the early stage of IAV infection, NP S9 and Y10 are dephosphorylated to facilitate NP nuclear import, and Y296 is phosphorylated to inhibit NP nuclear export, which is inverse during the late stage of infection (Zheng et al., 2015).

Phosphorylation levels of proteins are regulated by kinase-catalyzed phosphorylation and phosphatase-catalyzed dephosphorylation. There are quite a few studies related to the regulation of influenza virus replication by kinases. Activated human protein kinase C (PKC) family member PKCδ interacts with the polymerase subunit PB2 and regulates NP oligomerization and RNP assembly in the course of IAV infection (Mondal et al., 2017). Serine/threonine kinases polo-like kinases (PLK1, PLK3 and PLK4) and Akt kinase promote influenza virus replication (Hirata et al., 2014; Pohl et al., 2017). Also, JAK and Src kinase inhibitors (AG490 and Dasatinib) accelerate the nuclear import of IAV M1 (Wang et al., 2013). Hence, the kinases have influence on multiple steps of the viral life cycle. In this study, we demonstrated that tyrosine kinases Janus and Src catalyzed Y78 phosphorylation and regulated NP nuclear export during viral replication. Similarly, phosphorylation of Y10 and Y296 has also been reported to be involved in NP subcellular localization (Zheng et al., 2015), but which kinds of tyrosine kinases regulate their phosphorylation need to be further studied. At the same time, we found that tyrosine kinase Bcr/Abl inhibitor Imatinib suppressed NP phosphorylation at a similar level to tyrosine kinases Janus and Src inhibitor Dasatinib and AG490. However, Imatinib did not affect NP localization. It is indicated that the phosphorylation of other tyrosine residues might play different roles during virus infection. Moreover, which tyrosine residues (one or more residues) are regulated by tyrosine kinase Bcr/Abl is an interesting future direction.

To be the major structural component of vRNP, NP performs a vital function in the replication of influenza virus by nuclear-cytoplasmic shuttling (Whittaker et al., 1996b; Boulo et al., 2007; Li et al., 2015). NP on vRNP interacts with M1 and bridges the CRM1-NEP-M1 complex and viral polymerase heterotrimer during vRNP nuclear export (Akarsu et al., 2003; Shimizu et al., 2011). Here, we discovered that constitutive phosphorylation of NP Y78, which is mimicked by Y78E, affected the interaction of NP with CRM1, then suppressed singly expressed NP nuclear export. Moreover, the Y78E mutant also inhibited the nuclear export of M1, NEP, and PB1, indicating that NP Y78 phosphorylation might further block the nuclear export of NEP-M1-vRNP complex. However, whether NP phosphorylation-regulated CRM1-NP binding has influence on the nuclear export of vRNP complex still remains unknown.

Two NLSs on NP control the nuclear import of vRNP (Weber et al., 1998; Cros et al., 2005), and three nuclear export signals (NESs) on NP participate in the vRNP’s export into the cytoplasm (Yu et al., 2012). At the same time, importin α (Melen et al., 2003; Nakada et al., 2015) and Chromosome Region Maintenance protein 1 (CRM1) (Elton et al., 2001; Chutiwitoonchai et al., 2017) also take part in this process. The NES mutations usually result in the NP nuclear retention. Among them, NES3 (248-274) is crucial for virus growth, and its mutant virus cannot be packaged (Chutiwitoonchai et al., 2014). Besides, NES3 depends on CRM1, but NES1(24-49) and NES2 (183-197) do not (Yu et al., 2012). In our study, phosphorylation of Y78 was found to inhibit NP nuclear export by reducing the interaction of NP with CRM1, indicating that Y78 phosphorylation suppressed NES3-controlled NP nuclear export. However, Y78 does not locate at any NES of NP. These data suggest that the phosphorylation sites outside NES regions of NP can also regulate NP nuclear export *via* a CRM1-dependent pathway.

In conclusion, we identified the tyrosine phosphorylation site Y78 on NP and discovered that Y78 phosphorylation regulates the nuclear export and ubiquitination of NP, which is vital for the replication of IAV. Our data further expand the biological functions of NP phosphorylation and provide a potential target for the development of antiviral agents.

## Data Availability

The raw data supporting the conclusions of this manuscript will be made available by the authors, without undue reservation, to any qualified researcher.

## Ethics Statement

All embryonated chicken eggs experiments were approved by the Research Ethics Committee of Chinese Academy of Sciences and complied with the Beijing Laboratory Animal Welfare and Ethical Guidelines of the Beijing Administration Committee of Laboratory Animals.

## Author Contributions

WL conceived the study. LS and LC designed the experiments, analyzed the data, and wrote the manuscript. LC performed the experiments. WZ provided experimental technical guidance and results analysis. ML, XB, and WY helped with some experiments. JL, WF, and GG helped analyze the data and revise the manuscript.

### Conflict of Interest Statement

The authors declare that the research was conducted in the absence of any commercial or financial relationships that could be construed as a potential conflict of interest.
